# High‐latitude kelps and future oceans: A review of multiple stressor impacts in a changing world

**DOI:** 10.1002/ece3.10277

**Published:** 2023-07-04

**Authors:** Veronica Farrugia Drakard, Jordan A. Hollarsmith, Michael S. Stekoll

**Affiliations:** ^1^ Juneau Center, College of Fisheries and Ocean Sciences University of Alaska Fairbanks Juneau Alaska USA; ^2^ Alaska Fisheries Science Center National Marine Fisheries Service, National Oceanic and Atmospheric Administration Seattle Washington USA

**Keywords:** climate change, kelp, marine ecology, multiple stressors, phycology

## Abstract

Kelp forests worldwide are threatened by both climate change and localized anthropogenic impacts. Species with cold‐temperate, subpolar, or polar distributions are projected to experience range contractions over the coming decades, which may be exacerbated by climatic events such as marine heatwaves and increased freshwater and sediment input from rapidly contracting glaciers. The northeast Pacific has an extensive history of harvesting and cultivating kelps for subsistence, commercial, and other uses, and, therefore, declines in kelp abundance and distributional shifts will have significant impacts on this region. Gaps in our understanding of how cold‐temperate kelp species respond to climate stressors have limited our ability to forecast the status of kelp forests in future oceans, which hampers conservation and management efforts. Here, we conducted a structured literature review to provide a synthesis of the impacts of multiple climate‐related stressors on kelp forests in the northeast Pacific, assess existing knowledge gaps, and suggest potential research priorities. We chose to focus on temperature, salinity, sediment load, and light as the stressors most likely to vary and impact kelps as climate change progresses. Our results revealed biases in the existing literature toward studies investigating the impacts of temperature, or temperature in combination with light. Other stressors, particularly salinity and sediment load, have received much less focus despite rapidly changing conditions in high‐latitude regions. Furthermore, multiple stressor studies appear to focus on kelp sporophytes, and it is necessary that we improve our understanding of how kelp microstages will be affected by stressor combinations. Finally, studies that investigate the potential of experimental transplantation or selective cultivation of genotypes resilient to environmental changes are lacking and would be useful for the conservation of wild populations and the seaweed aquaculture industry.

## Graphical abstract

Kelp forests worldwide are threatened by both climate change and localized anthropogenic impacts. Here, we conducted a structured literature review to provide a synthesis of the impacts of multiple climate‐related stressors on kelp forests in the northeast Pacific, assess existing knowledge gaps, and suggest potential research priorities. Our results revealed biases in the existing literature toward studies investigating the impacts of temperature, or temperature in combination with light, and toward studies focussing on kelp sporophytes.

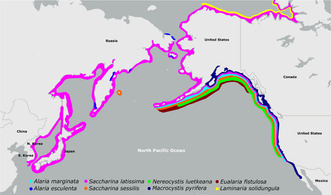

## 1 INTRODUCTION

Kelps are large brown macroalgae of the order Laminariales which form extensive underwater forests and have a widespread distribution across temperate and polar coastlines (Smale, 2019). These organisms are of fundamental importance to the coastal systems in which they occur and provide diverse ecological and economic benefits. The biogenic habitat they provide constitutes one of the most productive ecosystems on the planet, supports a rich associated biodiversity, and provides critical ecosystem services, including erosion control, nutrient cycling, and the provision of shelter and nursery areas for socioeconomically important fish species and multiple life stages of other marine species (Siddon et al., 2008; Smale et al., 2013; Steneck & Johnson, 2013; Teagle et al., 2017; Wernberg et al., 2019).

Kelp forests worldwide are coming under threat from the combined pressures of global climate change and localized anthropogenic impacts. Kelp forests are highly dynamic systems that are sensitive to changes in the physical, chemical, or biological characteristics of their environment (Smale et al., 2013). Point stressors such as coastal hardening (Marzinelli et al., 2011; Mayer‐Pinto et al., 2018), eutrophication (Strain et al., 2014; Tegner et al., 1995), herbivory (McPherson et al., 2021; Rogers‐Bennett & Catton, 2019), and changes in physicochemical factors (Lind & Konar, 2017; Schoch & Chenelot, 2004; Vettori et al., 2020) may lead to altered kelp forest function, changes in associated biota, localized declines in kelp abundances, and even local extinctions (Smale, 2019). Globally, rising sea surface temperatures and an increased frequency of marine heatwaves have led to widespread changes in kelp distributions (Diehl et al., 2021; Gordillo et al., 2022; Martínez et al., 2018; Smale et al., 2019; Tait et al., 2021), including range expansions for cold range edge species and range contractions for warm range edge species (Smale, 2019), with knock‐on effects for associated biodiversity and trophic exchanges (Schiel et al., 2014). These impacts are particularly pronounced in polar, sub‐polar, and cold‐temperate regions.

Rising sea surface temperatures have resulted in poleward shifts in species ranges, particularly for cold‐temperate species (Goldsmit et al., 2021; Wilson et al., 2019). Rates of warming in the Arctic are approximately four times higher compared to the rest of the globe, a phenomenon known as polar amplification (Rantanen et al., 2022). Therefore, it is likely that kelp species with current cold‐temperate, subpolar, or polar distributions will experience significant range contractions over the coming decades, which may be exacerbated by extreme climatic events such as marine heatwaves, potentially to the point of local extirpations or global extinctions (Thomsen et al., 2019; Wilson et al., 2019). Additionally, polar, subpolar, and cold‐temperate regions are susceptible to the effects of glacial melt, which contributes to decreased salinities and increased sedimentary deposits at glacial outflows, both of which have been shown to impact kelp physiology and the provision of ecosystem services (Lind & Konar, 2017; Picard, Johnson, & Côté, 2022; Traiger & Konar, 2017; Vettori et al., 2020). Glacial contraction is likely to be exacerbated by increasing sea surface temperatures, resulting in a higher rate of freshwater and sediment deposition into coastal environments. Although ongoing climate change will also result in pH effects, including ocean acidification, research suggests that these changes will have minimal to no impact on the physiology of kelp species (Fernández et al., 2015; Roleda et al., 2012). A deeper understanding of the dynamics of kelp forests in these cold‐water regions is fundamental to developing predictions of kelp functioning and distributions under future ocean conditions.

The northeast Pacific, and Alaska in particular, is likely to experience significant shifts in species distributions under warming conditions. This region represents the northern limit of distribution for several seaweed species which occur mainly to the south of Alaska (Stekoll, 2019), including *Alaria marginata*, several species of *Laminaria* and *Saccharina*, and the canopy‐forming kelps *Macrocystis pyrifera*, *Nereocystis luetkeana*, and *Eualaria fistulosa* (Stekoll, 2019). Humans have harvested kelp in the northeast Pacific since time immemorial, and current kelp harvest supports important subsistence and commercial food products and fisheries. *N. luetkeana*, for example, is harvested for processing into commercially available food products, and *M. pyrifera* is harvested as part of the roe‐on‐kelp fishery (Stekoll, 2019). Recently, there has been increasing interest and investment in the commercial cultivation of a few species, including *Saccharina latissima* and *A. marginata* (Stekoll et al., 2021). This is an emerging and potentially highly lucrative industry that is likely to experience significant impacts from the combined effects of global and local stressors on kelp ecology, physiology, and distribution.

Up to this point, much of the research involving the susceptibility of kelps to climate change and anthropogenic stressors has been focused on the impacts of rising sea surface temperatures and marine heatwaves, and a number of comprehensive reviews exist in this regard, particularly from the North Atlantic (Smale, 2019; Smale et al., 2013, 2019). Comparatively few reviews have been concerned with the northeast Pacific region (but see Hollarsmith et al., 2022). More recently, there has been increasing interest in the impacts of anthropogenic stressors on the microscopic stages of kelps, heretofore considered the “black box” of the kelp life cycle due to the difficulties involved in taking in situ measurements (Coelho et al., 2000; Martins et al., 2023; Veenhof et al., 2022); however, there is much left to be done in this regard and full consideration of how this may impact the economic benefits and environmental services of kelp forests is lacking.

This review aims to provide a synthesis of the potential impacts of multiple climate‐related stressors on kelp forests in Alaska and the northeast Pacific. We review the literature on responses of cold‐temperate, subpolar, and polar kelp species of this region to climate‐related stressors, considering impacts on both the macroscopic and microscopic stages of the life cycle and assessing whether the stressors in question have been tested singly or in combination. Subsequently, we identify key patterns emerging from the literature and consider the implications with regard to the persistence of cold‐adapted kelps under future ocean conditions. Finally, we identify the main knowledge gaps arising from this review and suggest potential research priorities over the coming decade.

## 2 REVIEW METHODS

**FIGURE 1:**
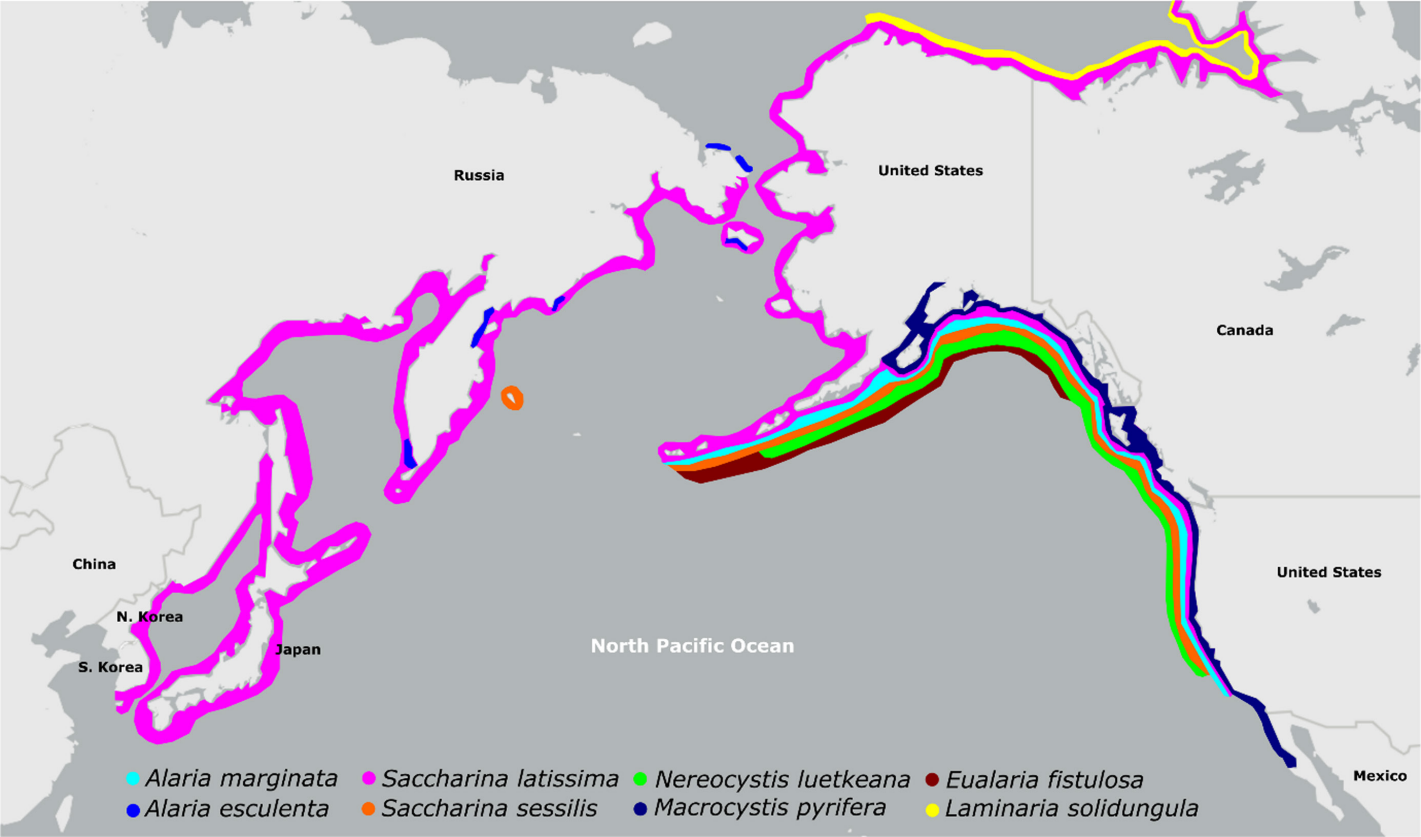
Map of the northern Pacific Ocean showing the approximate distributional ranges of the key species included in this review.

The studies included in this review were selected through a systematic literature search in Scopus, targeting climate and anthropogenic stressors likely to have the greatest impact on kelps in Alaska and the northeast Pacific region, defined here as the region between 30°N and 60°N, and between 120°W and 180°E (Table 1). As water temperatures increase, glacial contraction will decrease salinity and increase sediment load, which in turn attenuates light penetration. Temperature, salinity, sediment, light, and UV were, therefore, considered to be most likely to vary over the coming decades as a result of ongoing climate change and to directly impact kelp species, and were therefore selected a priori as stressors of interest. The genera *Saccharina*, *Alaria*, *Nereocystis*, *Eualaria*, and *Macrocystis* were considered to have the most ecological and commercial importance in the northeast Pacific region (Figure 1) and were therefore selected a priori as genera of interest. Our initial search resulted in a total of 1187 results, which were subsequently screened to determine their suitability for inclusion in this study. We excluded: (i) duplicate results, (ii) results that were outside of the scope of this review, (iii) studies conducted outside of the temperate to polar latitudes of the Northern Hemisphere, and (iv) reviews and meta‐analyses. Kelp populations outside of the region of focus are subject to different biological and environmental pressures, such as interspecific interactions and climatic patterns, and were therefore considered to be out of scope. After screening, a total of 193 studies were included in this review (Data S1).

**TABLE 1:** Search strings used for the systematic literature search on the impacts of climate‐related stressors on kelp species in the northeast Pacific.

Theme	Search string
By region	(laminariales OR kelp) AND (temperature OR salinity OR sediment OR light OR uv) AND (north) (laminariales OR kelp) AND (temperature OR salinity OR sediment OR light OR uv) AND (polar OR subpolar OR sub‐polar) (laminariales OR kelp) AND (temperature OR salinity OR sediment OR light OR uv) AND (arctic OR subarctic OR sub‐arctic) (laminariales OR kelp) AND (temperature OR salinity OR sediment OR light OR uv) AND (temperate)
By species of interest	(kelp) AND (temperature OR salinity OR sediment OR light OR uv) AND (saccharina) (kelp) AND (temperature OR salinity OR sediment OR light OR uv) AND (alaria) (kelp) AND (temperature OR salinity OR sediment OR light OR uv) AND (nereocystis) (kelp) AND (temperature OR salinity OR sediment OR light OR uv) AND (macrocystis) (kelp) AND (temperature OR salinity OR sediment OR light OR uv) AND (eualaria) (temperature OR salinity OR sediment OR light OR uv) AND (alaria AND marginata)

Each result was individually analyzed and assigned specific keywords or combinations of keywords (Table 2) according to the following criteria: (i) species of interest, (ii) stressor of interest, (iii) experiment type, (iv) single versus multiple stressor design, (v) stressor interactions, (vi) individual stressor effects, (vii) kelp life stage, (viii) response variable, and (ix) geographical region. As very few existing studies consider *A. marginata*, the north Atlantic/Arctic species *A. esculenta* was included in this review as the closest extant congener. This is also a cold‐adapted subcanopy species and is therefore expected to have similar ecological characteristics and will respond to climate variability in a similar way. These data were subsequently visualized in order to elucidate broad patterns and biases across this pool of studies.

**TABLE 2:** Thematic keywords assigned to each individual study included in this review.

Criterion	Keywords
Species of interest	*Alaria marginata*/*Alaria esculenta*, *Laminaria solidungula*, *Saccharina latissima*/*Saccharina sessilis*, *Macrocystis pyrifera*/*Macrocystis integrifolia*, *Nereocystis luetkeana*, *Eualaria fistulosa*, Others
Stressor of interest	UV, Temperature, Light, Physical, Sediment, Salinity, Others
Experiment type	Lab, Observational, Manipulative, Modeling
Single versus multiple stressor design	Single, Multiple
Stressor interactions	Complex, Unspecified, None, Additive, Antagonistic, Synergistic
Stressor effects	Unspecified, Tolerant, Positive, Negative, Neutral
Kelp life stage	Microstage, Macrostage
Response variable	Distribution, Physiology, Morphology, Biochemical, Others
Geographical region	Global, Arctic, Sub‐arctic, Temperate

## 3 RESULTS AND DISCUSSION

### 3.1 General trends and patterns in the literature

**FIGURE 2:**
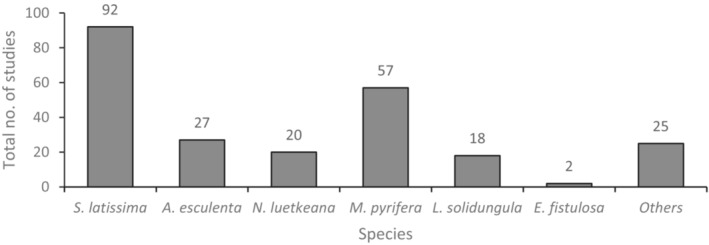
Total number of studies considering each of the major kelp species encountered in this review: *Saccharina latissima*, *Alaria esculenta*, *Nereocystis luetkeana*, *Macrocystis pyrifera*, *Laminaria solidungula*, *Eualaria fistulosa*, and others. ‘Others’ representing *Agarum* spp., *Chorda filum*, *Costaria costata*, *Egregia menziesii*, *Pleurophycus gardneri*, and *Pterygophora californica*.

Approximately 45% of the studies identified in this review are concerned with kelp populations in arctic or sub‐arctic regions, while 58% are concerned with populations in temperate regions. Most studies focus on one of two major species: *S. latissima* (48%) (formerly *Laminaria saccharina*) and *M. pyrifera* (30%) (Figure 2). This is not entirely unexpected; *M. pyrifera* is the most widely‐distributed kelp species on the planet, ranging from the temperate and subpolar coastlines of South America, the South Atlantic, and the Sub‐Antarctic islands up to the northeast Pacific (Mora‐Soto et al., 2020). *Saccharina latissima* has a similarly widespread distribution in temperate, polar, and subpolar regions of the northern hemisphere (Nielsen et al., 2014). Additionally, there is a long history of the commercial harvest of *S. latissima* in both the Pacific and Atlantic Oceans (Peteiro et al., 2016). Comparatively few studies focus on other species of interest in Alaska and the NE Pacific, such as *A. marginata* or *N. luetkeana* (10%). Most studies involving the genus *Alaria* focus on *Alaria esculenta* (14%), but even these are limited in number compared to *S. latissima* and *M. pyrifera*, as previously described. Both *A. marginata* and *N. luetkeana* are ecologically and commercially relevant in the NE Pacific—the latter is a major canopy‐forming species along Pacific coastlines and is harvested for commercial use (Springer et al., 2010; Stekoll et al., 2006), while *A. marginata* is a significant component of the mariculture industry in this region (Stekoll, 2019).

**FIGURE 3:**
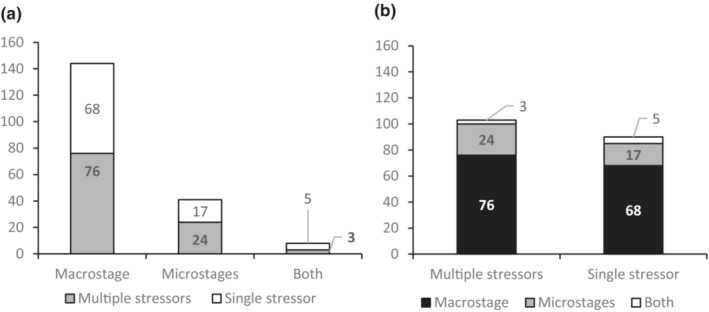
Number of studies included in this review that consider (a) the kelp macrostage, microstages, or both, and (b) multiple stressor systems or single stressors.

Approximately 21% of studies specifically investigate the responses of early life stages of kelps to stressor impacts (Figure 3a). As previously mentioned, these kelp microstages are difficult to study in the field (Coelho et al., 2000; Martins et al., 2023; Veenhof et al., 2022), and it is therefore only logical that most studies would focus on the sporophyte stage, as wild populations are readily located and present throughout most of the year. The majority of studies measure physiological responses to stress, including growth, survival, and reproductive responses.

In general, slightly more studies consider the impacts of multiple stressor systems (54%) than the impacts of single stressors (46%) (Figure 3b). The overwhelming majority focus on temperature (60%). This reflects climate priorities, as a rise in sea surface temperature and the frequency of marine heatwaves is likely to be the major climate‐related impact on marine systems over the coming decades (Smale, 2019; Smale et al., 2019). When stressors are considered in combination, most studies are concerned with temperature and light, indicating a focus on both climate priorities and those environmental factors which are most likely to have direct effects on kelp ecology and physiology. However, the prevalence of multiple stressor studies may reflect a growing awareness that the oceans constitute a system of interconnected components and influences, most of which are likely to be affected in complex ways by a changing climate. We consider the impacts on NE Pacific kelp species of the most salient stressors and stressor combinations arising from this review in the subsequent sections. The following stressors were encountered only infrequently in the literature over the course of this review, and have therefore not been included as a focus: interspecific and intraspecific competition, ocean acidification, geophysical variation, ice cover, heavy metal concentration, O_2_ concentration, and petroleum contamination.

**FIGURE 4:**
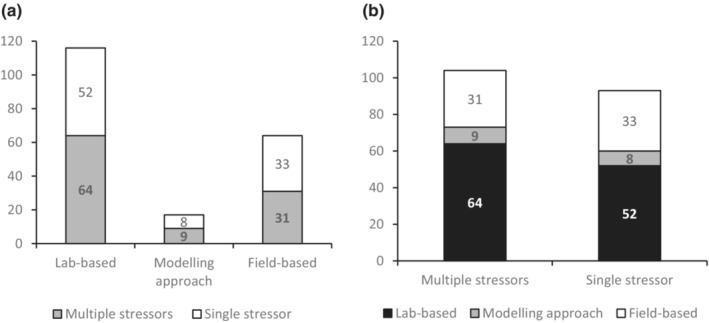
Number of studies included in this review that consider (a) lab‐based, modeling, and field‐based approaches, and (b) multiple stressor systems or single stressors.

Finally, it is worth considering the different experimental approaches encountered during this review. The majority of studies, a total of 116 (~60%), involved laboratory‐based experiments. A total of 64 studies (~33%) involved field‐based approaches, and only 17 studies (~9%) utilized computer‐based modeling approaches (Figure 4a). Across these categories, we observed a fairly even split between multiple‐stressor and single‐stressor approaches (Figure 4b).

### 3.2 Individual stressor impacts

#### 3.2.1 Temperature

A total of 117 studies consider the impacts of temperature either in isolation or in conjunction with other stressors. Of these, 73% report negative biological effects as a result of increasing temperature, while 11% report positive biological effects. This apparent discrepancy is a consequence of different temperature ranges being tested across different studies. In general, any variation in temperature outside of the optimum range for a given species—whether in colder or warmer conditions—will result in negative effects.

Temperature tolerance ranges for kelp sporophytes vary by species; for example, *S. latissima* sporophytes can survive temperatures between −1.5 and 23°C, while *A. esculenta* has a slightly lower upper survival limit of 21°C (Dieck, 1993). Increasing temperature beyond the optimum for a given species has a variety of impacts, including decreasing photosynthetic yield (Niedzwiedz et al., 2022), overall individual survival, and biomass and tissue strength in *S. latissima* (Simonson, Scheibling, & Metaxas, 2015), quality and commercial value in *S. latissima* and *M. pyrifera* (Lowman et al., 2022; Simonson, Metaxas, & Scheibling, 2015), phenotypic plasticity in *N. luetkeana* (Supratya et al., 2020), and spore development and settlement in *M. pyrifera* (Le et al., 2022). On the contrary, a rise in sea surface temperature is likely to favor kelp populations at the lower threshold of their thermal tolerances, resulting in overall positive biological effects and range expansions into northern regions by cold range edge species (Smale, 2019).

Impacts of temperature on kelp microstages are varied. The majority of studies report negative effects of increasing temperature (69%), with only 16% reporting positive effects. Oceanographic conditions during winter have been shown to be a good predictor of summer canopy cover of *N. luetkeana*; heatwave events in winter, which would impact nutrient availability for gametophytes and early sporophytes, led to sharp declines in canopy abundance during the following summer (García‐Reyes et al., 2022). Lind and Konar (2017) report that the early life‐history stages of Alaskan kelps experience significant impacts at elevated temperatures, including decreases in spore settlement concentration, spore germination, and germ tube growth. However, some degree of settlement and initial growth was still observed, suggesting that kelp microstages may be resilient to thermal stress to a certain extent (Lind & Konar, 2017). In *S. latissima* gametophytes, temperature has been shown to modulate the expression of sex‐biased genes, with female individuals exhibiting more marked responses than males—this may have implications for reproductive success of this species in a warming environment (Monteiro, Heinrich, et al., 2019). Responses of kelp microstages to thermal stress vary across populations within a species; for example, Hollarsmith et al. (2020) report developmental failure at the egg and sporophyte stages of *M. pyrifera* from high‐latitude California populations, whereas specimens from low‐latitude populations are able to produce sporophytes at elevated temperatures. This suggests that certain populations within a given species may exhibit a degree of thermal resilience, which would have important implications for the conservation of cold‐adapted species.

A warming ocean will likely result in significant changes in distribution for a number of kelp species, specifically range contractions for cold‐adapted species and range expansions for warm‐adapted species (Martínez et al., 2018). For any given species, the impacts of warming are likely to be more pronounced in trailing edge populations, which exist close to their thermal survival limits (Tait et al., 2021). Therefore, in colder regions, elevated temperatures are likely to disproportionately favor species with more temperate distributions. For example, *S. latissima* sporophytes from Norway exhibit an increase in growth and maximal yield for photosystem II fluorescence (Li, Monteiro, et al., 2020), and gametophytes of this species from this region are able to produce sporophytes at 15°C, while gametophytes of *A. esculenta* from the same region do not produce sporophytes at the same temperature (Park et al., 2017). Furthermore, it has been shown that sporophytes of *S. latissima* from Norway show no decrease in photosynthetic yield at 15°C (Andersen et al., 2013) and still have a measurable, albeit greatly reduced, photosynthetic yield at 20°C (Park et al., 2017), while sporophytes of *A. esculenta* have a photosynthetic yield of zero at the latter temperature (Park et al., 2017). The spores and gametophytes of both *M. pyrifera* and *N. luetkeana* experience significant negative impacts under elevated temperatures and hyposaline conditions, including decreased spore settlement concentration and germ tube growth, but these effects are not as pronounced for *Eualaria fistulosa*, a less widely‐distributed species (Lind & Konar, 2017). Furthermore, for *M. pyrifera* the upper thermal threshold for spore and germling development has been reported to be between 21.7 and 23.8°C (Le et al., 2022). This suggests that species such as *M. pyrifera* and *S. latissima* may experience range expansions into higher latitudes, whereas more cold‐adapted species such as *A. esculenta* and *A. marginata* will experience range contractions and may disappear from areas at their lower distribution limits. It is important to note, however, that species distributions will also be affected by oceanographic and hydrographic factors, and are therefore not simple to predict.

Heatwave scenarios and extended periods of elevated temperatures are likely to have long‐term impacts on kelp populations regardless of species. Nepper‐Davidsen et al. (2019) show that a 3‐week exposure to elevated temperatures results in significant decreases in growth rate and photosynthetic performance in sporophytes of *S. latissima*, although effects on mortality are limited. The authors suggest that these long‐term impacts may increase the susceptibility of kelp populations to other stressors. Additionally, Filbee‐Dexter et al. (2020) demonstrate that heatwave events during 2018 were linked to high rates of mortality for *S. latissima* on both sides of the North Atlantic. Maximum temperature anomalies exceeded lethal thresholds for this species both in southern Norway and southern New England, USA, resulting in mortality levels significantly higher than historical averages (Filbee‐Dexter et al., 2020). The authors indicate that these mortality rates if sustained over a longer period of time, could lead to persistent loss or local extinction of *S. latissima* in these areas (Filbee‐Dexter et al., 2020). Furthermore, multiple studies have indicated that persistent marine heatwaves render kelp forests vulnerable to subsequent stress events, including biotic pressures, and drive shifts from kelp forest ecosystems to sea urchin barrens and turf reefs with low primary productivity (McPherson et al., 2021; Rogers‐Bennett & Catton, 2019).

Overall, it appears that increasing temperature beyond the optimum will result in reduced germination success and growth rates for kelp microstages (Hollarsmith et al., 2020; Lind & Konar, 2017), along with increased mortality and decreases in abundance for the sporophyte stage (Filbee‐Dexter et al., 2020; Nepper‐Davidsen et al., 2019; Park et al., 2017). Local extirpations and range contractions for cold‐adapted species may follow, while warm‐adapted species are likely to experience increases in their global distributions (Martínez et al., 2018; Smale, 2019; Tait et al., 2021). In the region of focus of this paper, this suggests that *A. marginata* and *N. luetkeana* are more likely to experience range contractions, whereas *M. pyrifera* and *S. latissima* may expand their ranges. There is currently a lack of information regarding how *E. fistulosa* responds to changes in temperature, and therefore predictions of how this species will fare in future oceans are less straightforward. Long‐term thermal stress increases the susceptibility of kelp populations to other stressors, and so the temperature is likely to interact with other climate‐related stressors in complex ways.

#### 3.2.2 Salinity

Salinity is considered as a stressor in only 26 (14%) of the studies included in this review. Changes in environmental salinity are usually associated with estuarine habitats and riverine influences, so salinity is not generally thought of as a climate stressor. However, a rise in global temperatures is likely to increase the rate of glacial melt and lead to changes in salinity in glacially‐influenced environments, particularly in polar and subpolar regions. Furthermore, precipitation during winter will be more likely to fall as rain rather than snow, resulting in more rapid runoff and longer periods of consistent freshwater input into the coastal environment, which will have an impact on the ecology of high‐latitude kelp forests.

Most studies focus on *S. latissima* (69%) or *A. esculenta* (27%), and in many cases, these are multiple stressor designs investigating the combined impacts of temperature and salinity (Diehl et al., 2020; Fredersdorf et al., 2009; Li, Monteiro, et al., 2020; Lind & Konar, 2017; Monteiro et al., 2021; Monteiro, Li, et al., 2019) or between salinity and irradiance (Muth et al., 2021; Springer et al., 2017; Spurkland & Iken, 2011b). In general, stress induced by hyposaline conditions appears to increase the susceptibility of kelps to extreme temperature changes (Diehl et al., 2020; Lind & Konar, 2017), but no interactions with irradiance are reported.

Hyposaline conditions (< 20 psu) are associated most often with reduced rates of photosynthesis (Karsten, 2007; Li, Monteiro, et al., 2020; Monteiro, Li, et al., 2019; Spurkland & Iken, 2011b) and the loss of photosynthetic pigments in *A. esculenta*, *S. latissima*, and *L. solidungula* (Karsten, 2007). However, other studies have reported declines in *S. latissima* sporophyte growth rates (Monteiro et al., 2021), and gametophyte growth rates and spore settlement of *N. luetkeana* and *S. latissima* (Lind & Konar, 2017). Vettori et al. (2020) demonstrated significant changes to the morphological and mechanical properties of *S. latissima* from Scotland after an hour of exposure to freshwater, including increased blade flexibility and toughness and a reduction in surface area; this has implications for the commercial cultivation of this species, as any changes to tissue properties will impact product quality and value. Kelp gametophytes appear to be particularly susceptible to hyposaline stress—Muth et al. (2021) show that while sporophytes of *Laminaria solidungula* growing off the north coast of Alaska are able to survive in hyposaline conditions (10 and 20 psu), gametophytes of the species are unable to produce more sporophytes due to a combination of zoospore loss and gametophyte germination failure. Therefore, hyposaline (<20 psu) conditions negatively impact sexual reproduction in this species, which could result in range contractions, local extirpations, or reduced genetic diversity.

Overall, responses to changes in salinity appear to be species‐specific (Bruhn et al., 2017) and population‐specific within a given species (Monteiro, Li, et al., 2019). Salinity tends to be much more variable than temperature along coastlines, with localized extremes associated with estuaries and glacial input, and there is evidence that specific populations have adapted to these conditions and exhibit genetic and phenotypic differences (Møller Nielsen et al., 2016; Spurkland & Iken, 2012). This fact suggests that certain kelp populations may be more resilient to climate‐induced changes in salinity, and bears further investigation.

#### 3.2.3 Light and UV

The impacts of irradiance are considered from two major perspectives in the literature reviewed here: the effects of photosynthetically active radiation (PAR, 400–700 nm) were considered in a total of 64 studies (33%), and the effects of ultraviolet radiation (UV‐A and UV‐B, 100–400 nm) were considered in a total of 29 studies (15%). Exposure of marine organisms in polar regions to both these types of radiation is expected to increase in frequency and duration over the coming decades due to continued stratospheric ozone depletion over the poles (Müller et al., 2012). However, there is evidence that increased sediment load protects kelp forests from higher light intensities to a degree (Roleda et al., 2008), and therefore an increased rate of glacial contraction due to ongoing climate change may serve to mitigate increases in both PAR and UV radiation.

In general, intensities of PAR below the optimum required for photosynthesis result in decreased growth rates and has knock‐on effects for overall kelp abundances (Bonsell & Dunton, 2018; Kavanaugh et al., 2009; Mabin et al., 2019; Spurkland & Iken, 2011b). However, intensities of PAR beyond optimal levels and increasing intensity of UV wavelengths also have negative effects on kelp species, including cellular, enzymatic, and molecular damage, decreased growth rates, changes in DNA expression, and photo‐oxidative stress in *S. latissima* (Bruhn & Gerard, 1996; Heinrich et al., 2012, 2015; Roleda et al., 2006b) and photoinhibition in *M. pyrifera* and *N. luetkeana* (Clendennen et al., 1996; Mabin et al., 2019; Poulson et al., 2011). The majority of these effects are the result of light‐induced damage to photosystems I and II and chloroplast membranes (Bruhn & Gerard, 1996; Holzinger et al., 2011). However, in many cases, the magnitude of these effects varies by population and depends on exposure time and specific wavelength (Bruhn & Gerard, 1996; Roleda, 2009; Roleda et al., 2006a).

The literature suggests that kelp microstages are more susceptible to higher irradiance than sporophytes (Müller et al., 2008; Roleda et al., 2005, 2006a). Light intensity and exposure time control sporulation, spore settlement, and germination in *M. pyrifera* (Graham, 1996), and the growth and development of gametophytes in *M. pyrifera* and *S. latissima* (Huovinen et al., 2000; Makarov & Voskoboinikov, 2001). Specifically, studies have found that increasing intensity of PAR (>20 μmol/m^2^/s) causes photoinhibition in spores of *S. latissima*, and UV‐A and UV‐B wavelengths have a significant additional effect; spores did not recover from exposure times of over 8 h to UV‐A and over 4 h to UV‐B (Roleda et al., 2005; Wiencke et al., 2000, 2004). This loss of zoospore viability is thought to be the result of photodamage to both DNA and the photosynthetic apparatus (Wiencke et al., 2000). These impacts are often population‐specific, with evidence of ecotypic differences across the distribution range of particular species. For example, Müller et al. (2008) report that while UV‐B consistently inhibits spore germination in two separate populations of *S. latissima*, the effects of UV‐A vary by population and are modulated by temperature. Gerard (1990) shows ecotypic differences in the photosynthetic capacity and photoacclimation responses of *S. latissima* (as *Laminaria saccharina*) from three different populations growing in different ambient light regimes. This suggests that certain species or populations may be quicker to adapt to changes in light intensity or exposure time than others (Palacios et al., 2021), which has implications for the ecology and persistence of cold‐temperate kelps in a changing climate.

Overall, kelp microstages appear to be more sensitive to irradiance levels than the sporophyte stage, and variations in light penetration will impact the timing of sporulation and germination of both spores and gametophytes (Graham, 1996; Huovinen et al., 2000; Makarov & Voskoboinikov, 2001; Müller et al., 2009; Roleda et al., 2005, 2006a; Wiencke et al., 2000, 2004). Light intensities above optimal levels result in photodamage and decreased growth rates in sporophytes (Bruhn & Gerard, 1996; Clendennen et al., 1996; Heinrich et al., 2012; Mabin et al., 2019; Poulson et al., 2011; Roleda et al., 2006b), while light intensities below optimal levels will also lead to decreased growth rates and higher mortalities (Bonsell & Dunton, 2018; Kavanaugh et al., 2009; Mabin et al., 2019; Spurkland & Iken, 2011b). Regions of the northeast Pacific coastline that are subject to significant glacial influence will experience decreased light penetration in the coming years as climate change progresses and the rate of glacial melt increases (Roleda et al., 2008). Kelp forests in these areas are likely to exhibit reduced abundances and growth rates as a result of impacts on photosynthesis and the timing of sporulation and germination for microstages. Susceptibility to variations in light intensity varies both by species and by population (Bruhn & Gerard, 1996; Gerard, 1990; Palacios et al., 2021; Roleda, 2009; Roleda et al., 2006a)—for example, *S. latissima* displays significant ecotypic differences across its distributional range and may be better able to adapt to these changes.

#### 3.2.4 Sedimentation

Only 11 studies (6%) consider the impacts of sediment load either directly or indirectly. This can be a challenging environmental variable to investigate; the type and degree of sedimentation to which kelp beds are exposed tends to be a highly localized phenomenon, and sediment dynamics are difficult to accurately recreate in lab‐based studies. However, sedimentation does have a number of impacts on kelp beds and is relevant in areas subject to glacial and riverine influence. Glaciers discharge freshwater and terrestrial sediments into coastal environments, both of which alter local conditions in a number of ways and impact the structure and function of kelp forests (Spurkland & Iken, 2011a). Increased sediment load attenuates light penetration, thereby affecting photosynthesis and productivity, and also physically impacts kelp through sediment scour (Aumack et al., 2007; Picard, Johnson, Ferrario, et al., 2022).

The evidence suggests that in the short term, cold‐temperate kelp species are able to acclimate to and recover from increased sediment loads in the water column. Roleda and Dethleff (2011) show that short‐term increases in sediment loads have no impact on *S. latissima*, while Shaffer and Parks (1994) report that a landslide in Puget Sound initially reduced abundances in beds of *N. luetkeana*, which subsequently appeared to recover. Transient increases in sedimentation may even have positive effects by providing some degree of UV attenuation and protecting kelp tissues from photodamage (Roleda et al., 2008).

On the contrary, consistent and long‐term increases in sedimentation are associated with kelp bleaching, loss of photosystem II function, and tissue decay, all of which can be attributed to increased light attenuation under higher sediment loads (Roleda & Dethleff, 2011). Sediment scour is particularly influential in determining the survival and success of kelp microstages, especially during settlement and attachment to the substratum. The literature indicates that scouring effects and burial strongly inhibit spore germination in *S. latissima* and *A. esculenta* (Picard, Johnson, & Côté, 2022), gametophyte survival in *S. latissima* (Traiger & Konar, 2017), and sporophyte germination and growth in *M. pyrifera*, *S. latissima* and *A. esculenta* (Muth et al., 2021; Zacher et al., 2016). Interestingly, however, Picard, Johnson, and Côté (2022) and Picard, Johnson, Ferrario, et al. (2022) show that increased sediment loads actually improve spore attachment in *S. latissima*, and suggest that this is due to the chemotactic responses of spores to nutrients carried by sediment granules. Responses to sedimentation are therefore complex and likely to be species‐specific. Indeed, *S. latissima* is considered to be relatively tolerant to variations in turbidity when compared to *A. esculenta* or *N. luetkeana* (Picard, Johnson, & Côté, 2022; Traiger & Konar, 2017), and may gain a competitive advantage from an increase in sediment loads in glacially‐influenced waters.

In general, increased sediment load will lead to decreased light penetration and, consequently, decreased growth rates and tissue decay in sporophytes. Scouring effects and burial by increased sediment loads will severely impact the survival and success of kelp microstages, by inhibiting spore germination, gametophyte survival, and sporophyte germination. As previously discussed, glacially‐influenced areas will experience increased turbidity and therefore increased scouring and decreased light penetration under future climate conditions. These scouring and shading effects are likely to impact kelp abundances in these areas by directly affecting the growth of sporophytes and the survival of microstages. Once again, *S. latissima* has been shown to be relatively tolerant to variations in turbidity and is likely to persist in these areas, potentially outcompeting species such as *A. marginata* and *N. luetkeana*.

#### 3.2.5 Nutrients

The influence of nutrient supply on kelps was considered in 29 (15%) of the studies included in this review. Nutrient availability can be a limiting factor for kelp growth and plays a role in determining the limits of distribution of some species (Strong‐Wright & Taylor, 2022). However, nutrient fluxes tend to interact significantly with other environmental conditions; for example, nutrient availability varies substantially by temperature, and we have already discussed how nutrients may be carried by sediment granules (Picard, Johnson, & Côté, 2022). Indeed, nutrient supply is often inextricably linked to large‐scale, complex climatic processes (Dayton et al., 1992; Ladah & Zertuche‐González, 2022). The impacts of ongoing climate change on these processes are not straightforward to determine, and therefore it is difficult to predict how nutrient supply might influence kelp forests in the near future. Furthermore, other anthropogenic activities—such as agriculture—often impact nutrient availability on a local scale, and, therefore, nutrient supply tends to be highly spatially and temporally variable.

The influence of nutrient supply on kelp forests is complex and tends to depend on the nature of the ecosystem and the types of interspecific interactions present. For example, an increase in nutrient supply may favor fast‐growing filamentous algal species rather than large habitat formers such as kelps, and therefore eutrophication tends to drive shifts from kelp forests to less productive turf reef systems (Moy & Christie, 2012). On the contrary, studies have recorded sharp declines in kelp abundances associated with decreased nutrient availability as a result of changes in large‐scale ocean processes (Berry et al., 2021; García‐Reyes et al., 2022). Stekoll et al. (2021) suggest that nutrients added to the water in the late summer may improve *M. pyrifera* survival through the dark winters in southeast Alaska. An increased nutrient supply has been shown to result in higher growth rates (Dean & Jacobsen, 1984; Jevne et al., 2020) and higher thermal tolerances in several species (Fernández et al., 2020; Gerard, 1997; Schmid et al., 2020), while nitrate limitation decreases photosynthetic capacity in *M. pyrifera* (Umanzor et al., 2021).

Nutrients also play a significant role in controlling the kelp life cycle. Carney and Edwards (2010) show that while gametophytes of *M. pyrifera* are able to grow under conditions of almost no nitrate availability, nitrates and trace amounts of other nutrients are necessary for gametogenesis. Therefore, the absence of nitrates effectively delays the production of gametes. Nitrate levels have also been found to impact sporophyte recruitment and survival (Deysher & Dean, 1986; Hernández‐Carmona et al., 2001).

### 3.3 Multiple stressor systems and impacts

Once again, most of the studies included in this review that consider more than one stressor included temperature. The most frequently investigated stressor combination is temperature with light (27 studies), followed by temperature with nutrients (22 studies), and temperature with salinity (14 studies). Of the multiple stressor studies included here, 45% do not report any interaction between the stressors involved. When interactions are reported, these are most often synergistic at 25% of all multiple stressor studies, while antagonistic interactions are reported 14% of the time, and additive interactions are reported 3% of the time. The majority of studies considered the sporophyte stage (77%), while the rest focused on the kelp microstages. In this section, we will discuss the most frequently investigated stressor combinations, namely temperature/light, temperature/nutrients, and temperature/salinity.

With regards to temperature and light, when interactions are reported, these are most often synergistic between high temperature and low light, suggesting that deleterious effects on kelps are at their maximum under conditions of high temperature and low light intensities. Gordillo et al. (2022) report that losses in biomass for cold‐adapted kelp species cultured under continuous darkness are enhanced when the temperature is increased from 3 to 8°C. Additionally, after being cultured in continuous darkness at 8°C, *A. esculenta* and *S. latissima* are unable to resume growth and photosynthesis upon illumination (Gordillo et al., 2022). It is important to note, however, that the conditions as described in Gordillo et al. (2022) are extremes, and kelp forests in Alaskan waters are unlikely to experience total darkness as a result of increased turbidity. Furthermore, temperature‐induced loss of *M. pyrifera* during a marine heatwave event was found to be more significant in turbid conditions (Tait et al., 2021). It is thought that this phenomenon is the result of metabolic stimulation under elevated temperatures, resulting in kelps metabolizing their storage compounds more quickly and being unable to replace them in darkness or under reduced light conditions (Li, Scheschonk, et al., 2020). This has significant implications for the survival of kelp forests through and beyond the polar winter under future climate scenarios. Certain species, such as *S. latissima*, may compensate for metabolic stimulation by repressing transcriptomic activities related to energy‐demanding processes under elevated temperature conditions (Li, Scheschonk, et al., 2020). Indeed, this species exhibits significantly altered gene expressions at higher temperatures and even more so at higher temperatures under high light intensities, suggesting that this combination causes the most metabolic stimulation (Heinrich et al., 2015).

Changes in light intensity may compensate for temperature‐induced stress in kelps and vice‐versa. Increased light during the reproduction and development of microstages of *A. esculenta* has been shown to improve sporophyte survival at elevated temperatures (Martins et al., 2022). Conversely, a moderate increase in temperature has been shown to decrease photoinhibition under high light intensities and improve the recovery of photosynthesis in *A. esculenta* and *S. latissima* (Roleda, 2009). It is important to note, however, that increasing temperatures beyond the optimum for any kelp species have consistently negative impacts on photosynthetic recovery.

In terms of temperature in combination with nutrients, once again most studies do not report any interactions. When these are reported, the majority are synergistic wherein the most stressful conditions for kelps involve high temperatures and nutrient concentrations either below or above optimum levels. These interactions frequently involve large‐scale oceanographic processes such as the El Niño‐Southern Oscillation (ENSO); studies have shown that high temperatures and changes in upwelling patterns during ENSO events cause nutrient‐limited conditions over winter, reducing summer canopy cover of *N. luetkeana* (García‐Reyes et al., 2022). This indicates that kelp microstages may be particularly vulnerable to changes in temperature and nutrient availability. Umanzor et al. (2021) report that a combination of high temperatures and limited nitrates decreases the photosynthetic capacity of *M. pyrifera* juvenile sporophytes, and limits their photosynthetic recovery. In contrast, high temperatures have also been found to interact with eutrophication to synergistically drive shifts from kelp forests to less productive systems (Moy & Christie, 2012). Similarly to the interaction between light and temperature, changes in nutrient availability may compensate for temperature‐induced stress, as adequate nutrient supply has been shown to increase thermal tolerances in multiple cold‐temperate kelp species (Fernández et al., 2020; Gerard, 1997; Schmid et al., 2020).

Few studies report interactions between temperature and salinity. However, Monteiro, Heinrich, et al. (2019) and Monteiro, Li, et al. (2019) show that temperature modulates the impacts of a hyposaline environment on *S. latissima*, as genes related to photosynthesis and pigment synthesis are repressed at low temperatures in hyposaline conditions. This phenomenon appears to be ecotype‐specific, as temperate populations exhibit the most transcriptomic changes at low temperatures and hyposaline conditions, while cold‐adapted populations exhibit most changes at higher temperatures (Monteiro, Li, et al., 2019). Therefore, certain populations may be resilient to future changes in temperature and salinity, and approaches to kelp conservation may benefit from the identification and propagation of specific genetic profiles within the wider species distributions.

Kelp microstages are once again particularly vulnerable to combined changes in salinity and temperature. Lind and Konar (2017) report decreased spore settlement and gametophyte growth for both *N. luetkeana* and *S. latissima* under elevated temperatures and hyposaline conditions, although both species exhibit some resilience to these stressors. In *A. esculenta*, the germination capacity of spores is reduced under low salinities and extreme temperatures, but the temperatures tested thus far are probably not ecologically realistic under future climate scenarios, and the species appears to be mostly resilient to differing salinities under ecologically relevant temperature conditions (Fredersdorf et al., 2009).

## 4 CONCLUSIONS AND RECOMMENDATIONS

In this review, we have given an overview of the state of current knowledge regarding the impacts of climate‐related stressors on high‐latitude kelp species, as well as interpretations of how these kelp forests might respond to a changing ocean over the coming decades. It is clear that the main focus of the field thus far has been on changing temperatures and ocean warming, and while some questions remain, we now have a reasonable understanding of how this will impact kelp forest distributions and ecology going forward. Other climate‐related stressors have not received a similar level of attention, and our understanding of these is still lacking. For polar and subpolar regions in particular, further research is needed on the impacts of changing salinities and sedimentation rates on kelp species in areas subject to glacial influence. Most studies considered in this review were laboratory‐based, and therefore more work needs to be focussed on field‐based and modeling approaches.

An increasing number of studies are considering how combinations of multiple stressors will impact cold‐temperate kelp ecology (Gordillo et al., 2022; Heinrich et al., 2015; Li, Scheschonk, et al., 2020; Tait et al., 2021; Umanzor et al., 2021). This is a much more realistic representation of natural systems, and we would recommend that future investigations consider more varied combinations of stressors. In particular, salinity and sediment load are likely to vary in conjunction with one another in glacially‐influenced regions, and studies considering these two stressors in combination are lacking. Temperature is most frequently included in multiple stressor studies, but few investigations combine temperature with variations in salinity (Monteiro et al., 2021) or sedimentation—these combinations would provide invaluable information regarding the future of kelp forests in polar and subpolar regions in a warming climate.

Furthermore, while the studies in this review were nearly evenly split between those considering the sporophyte stage and those considering kelp microstages, the multiple stressor studies specifically appear to be biased toward kelp sporophytes. Kelp microstages appear to be very vulnerable to changes in environmental conditions (García‐Reyes et al., 2022), as these control if and when each stage proceeds to the next, and therefore the overall reproductive success of the kelp species in question. Therefore, it is necessary that we improve our understanding of how kelp microstages will be affected by stressor combinations in a changing climate. In structuring these multiple stressor investigations, researchers should ideally consider ecologically relevant stressor levels and combinations, with a view to how real‐world conditions are likely to change in the coming years.

Finally, several studies included in this review have indicated that several kelp species exhibit some degree of inherent resilience to changing environmental conditions (Hollarsmith et al., 2020; Palacios et al., 2021). This appears to be related to genetic variation within kelp species, wherein local adaptation, environmental conditioning, and/or epigenetic responses have resulted in ecotypes specifically resilient to certain environmental conditions (Gerard, 1990; Møller Nielsen et al., 2016; Monteiro, Li, et al., 2019; Müller et al., 2008; Spurkland & Iken, 2012). This may provide a path toward future‐proofing kelp forests in climatically vulnerable regions through active interventions. Studies that investigate the potential of experimental transplantation or selective cultivation of resilient genotypes would be useful both with a view toward conservation of wild populations and for increasing efficiency and economic return in a mariculture setting.

In terms of kelp forests in Alaska, based on the trends observed throughout this review, we would expect *S. latissima* to expand its distributional range throughout the Alaskan region. This species is relatively warm‐adapted (Andersen et al., 2013; Li, Monteiro, et al., 2020) and is also resilient to changes in sediment load and light penetration (Picard, Johnson, & Côté, 2022; Picard, Johnson, Ferrario, et al., 2022; Roleda & Dethleff, 2011; Traiger & Konar, 2017), and will therefore be able to persist in warming waters and glacially‐influenced areas. *Macrocystis pyrifera* is also likely to expand northwards and may come to dominate the high‐latitude subtidal in wave‐influenced areas (Hollarsmith et al., 2020; Le et al., 2022). As cold‐adapted species, *E. fistulosa* and *A. marginata* are likely to experience range contractions and may disappear from some regions of the Alaskan coastline entirely (Park et al., 2017).

## AUTHOR CONTRIBUTIONS

**Veronica Farrugia Drakard:** Conceptualization (equal); data curation (lead); formal analysis (lead); funding acquisition (equal); investigation (lead); methodology (lead); visualization (lead); writing – original draft (lead). **Jordan A. Hollarsmith:** Conceptualization (equal); funding acquisition (equal); supervision (equal); writing – review and editing (equal). **Michael S. Stekoll:** Conceptualization (equal); funding acquisition (equal); supervision (equal); writing – review and editing (equal).

## Supporting information


Data S1

## Data Availability

Data sharing is not applicable as no new data were created or analyzed in this study.
